# Laboratory markers as predictors of the outcome of resuscitation; a pilot study

**DOI:** 10.1186/1753-6561-6-S4-O29

**Published:** 2012-07-09

**Authors:** EC Reynolds

**Affiliations:** 1 University of Bristol, UK

## Introduction

Deciding when not to attempt resuscitation (DNAR) is problematic for many reasons but not least because of the difficulty in predicting the outcome in any given individual. “Futility” is often used as a justification for DNAR, but it lacks precision and is prone to subjectivity. Predictive scoring systems might avoid unnecessary pessimism and also minimise truly futile attempts which are distressing and undignified. Existing scores have a high specificity and low sensitivity, and may have useful negative predictive value but they are based on trials involving fewer than 200 patients. Sepsis and renal impairment appear to indicate a poor outcome but no studies have examined biochemical indicators of infection and inflammation (elevated C reactive protein (CRP) or white cell counts (WCC)). This feasibility study examined the relationship between elevated CRP, WCC, and creatinine and poor outcome of CPR.

## Methods

The study was performed retrospectively on information submitted to the national resuscitation audit database on CPRs between July 1st and Dec 31st 2010 in a large UK teaching hospital. Creatinine, CRP and WCC values up to 72 hours pre-arrest were analyzed. The study covered only ward-based arrests and excluded emergency department, intensive and coronary care units.

## Results

Data for 56 arrests were available in the study period. 8 patients survived to discharge (14.3%) and of the 48 who died (85.8%), 36 failed initial CPR, 8 died within 24 hours, and 4 died later in hospital. Data are presented as median values with ranges.

**Table 1:** Data for 56 arrests

Numbers	Survivors = 8 (14.2%)	Non-survivors = 48 (85.8%)
Age (years)	77.5 (48-94)	79.5 (35-98)
CRP (mg/L)	50.7 (2.6-147.7)	48.1 (2.4-278.3)
WCC (x109/L)	9.0 (4.1-19.3)	10.75 (4.2-18.3)
Creatinine (mmol/L)	80.0 (26-193)	106 (26-677)

Only 4 out of the 56 undergoing CPR had a CRP of less than 10.

## Conclusions

There was no correlation between age, creatinine, CRP, or WCC on the outcome of CPR suggesting these should not be major factors influencing DNAR decisions. However this study only involved small numbers and was a feasibility study for a larger analysis of around 1500 CPR attempts which will aim to construct and later validate a predictive scoring system.

